# Study on the changes and influencing factors of depression in Chinese women with cancer: an analysis based on CHARLS panel data

**DOI:** 10.3389/fpubh.2024.1485196

**Published:** 2025-01-16

**Authors:** Na Wang, Mengran Chang, Shidong Liu, Bing Chen

**Affiliations:** ^1^Gynecology Department, First Affiliated Hospital of Bengbu Medical University, Bengbu, China; ^2^China Cooperative Research Institute, Anhui University of Finance and Economic, Bengbu, China; ^3^School of Languages and Media, Anhui University of Finance and Economics, Bengbu, China

**Keywords:** women with cancer, depression, development trends, influencing factors, panel stochastic trend model

## Abstract

**Background:**

The social problems caused by depressive disorders and psychological behaviors in women are increasingly prominent, with extreme incidents occurring from time to time. Therefore, the issue concerning “how to prevent and resolve the risk of depression in women” is gaining significant attention across various sectors. However, previous studies have largely focused on teenage girls, perimenopausal women, or women during pregnancy and the postpartum period, neglecting the adverse effects of major diseases, which is detrimental to enhancing the psychological well-being of women with cancer.

**Objective:**

Based on cancer impacts, this study aims to clarify the depressive state, development trends, and influencing factors in Chinese women with cancer, a group particularly susceptible to depression, to provide decision-making references for improving their psychological well-being.

**Methods:**

Using follow-up panel data from five waves of China Health and Retirement Longitudinal Study (CHARLS), Chinese women with cancer who had complete observed values were selected as research subjects. After data cleaning, a balanced short panel dataset containing 1,030 samples was obtained. The depression characteristics and development trends in women with cancer were analyzed using the panel statistical method, and the influence of socio-demographic factors was examined using a panel stochastic trend model.

**Results:**

The overall depression of Chinese women with cancer has deteriorated, putting them at risk of moderate depressive disorder. The development trend shows an inverted “U” curve, with significant differences observed among various groups based on urban and rural residency, educational levels, and regional locations. Specifically, urban women with cancer show milder depressive symptoms than those in rural areas, and women in the eastern region, those with higher educational levels, and those with non-reproductive system cancers show milder depressive symptoms than those in the central and western regions, those with lower educational levels, and those with reproductive system cancers. Regression analysis indicates that socio-demographic factors significantly influence depression in women with cancer. Among these influencing factors, age, having a spouse, high educational level, good performance in the activity of daily living (ADL), frequent visits to neighbors, and regular exercise are protective factors against depression, while diabetes, dyslipidemia, frequent outpatient visits, frequent hospitalizations, smoking, and low life satisfaction are risk factors for depression.

**Conclusion:**

The depressive state among Chinese women with cancer is alarming. In view of this, there is an urgent need for introducing more supportive policies aimed at improving psychological health, developing tailored measures to address the specific needs of different groups, intensifying efforts to standardize the diagnosis and treatment of depression, particularly for those in rural areas, central and western regions, and among individuals with lower educational levels. Additionally, the role of social participation activities, such as visiting neighbors and exercising, should be given full play to alleviate depressive symptoms.

## 1 Introduction

Cancer is one of the major public health problems facing the world, which is a disease characterized by abnormal cell growth within the body that invades surrounding tissues or spreads to other parts of the body, medically known as malignant tumors. According to the latest data from the International Agency for Research on Cancer (IARC), the global number of new cancer cases in 2022 is close to 20 million, with 9.8 million cancer deaths. 49.2% of new cases and 56.1% of deaths from malignant tumors occur in Asia, with incidence and prevalence rates continuing to rise, putting huge pressure on the healthcare systems and social development of countries (1). In China, the situation of cancer incidence is also significant, becoming one of the leading causes of death (2). Despite some progress in early screening and treatment of cancer with medical technology, patients still face many unresolved issues during the treatment process, especially in terms of mental health challenges. Research shows a strong correlation between physical health and depressive states (3), especially among female cancer patients, who often face negative body perceptions and self-image issues during treatment. The interaction between the two can easily trigger or exacerbate depressive emotions, leading to social withdrawal and decreased interests, seriously affecting the quality of life and treatment outcomes (4). Therefore, focusing on the mental health of cancer patients, exploring the causes of their psychological issues, and coping strategies has become an urgent and important issue to address.

At the same time, with ongoing social development and the passage of time, the incidence of abnormal psychological behaviors and common mental disorders in China has been rising annually, becoming a significant public health and social problem that demands attention and resolution as part of advancing common prosperity in the new era. As one of the most prevalent mental disorders, depression, characterized by its high prevalence, prolonged treatment cycles, and high recurrence rates, imposes a heavy burden and wreaks havoc on individuals, families, and society, gradually evolving into the leading cause of disability worldwide (5) and the second-largest source of disease burden in China (2). Moreover, women are at a higher risk of depression compared to men, due to physiological stress factors such as menstruation, pregnancy, and perimenopausal changes, as well as the impact of family roles. The prevalence of depressive disorders in women is about 1.5 to 2 times higher than in men, with over 90% of female patients not receiving timely professional treatment (6, 7). The social problems caused by depressive disorders and psychological behaviors in women are becoming increasingly prominent, with extreme incidents occurring from time to time. Therefore, the issue concerning “how to prevent and resolve the risk of depression in women” is gaining significant attention across various sectors. In this context, extensive research on the manifestations, pathogenesis, and influencing factors of depression in women has been conducted by scholars both in China and from abroad, yielding fruitful results.

Clinical studies suggest that depression is one of the chronic psychiatric illnesses with high prevalence and high recurrence rates, typically presenting with symptoms such as persistent low mood, abnormal somatic characteristics, and varying degrees of cognitive and behavioral changes. In severe cases, it can lead to extreme outcomes such as pessimism, self-harm, and suicide (8). Epidemiological studies reveal that most female patients can effectively alleviate their symptoms and gradually return to normal functioning after standardized antidepressant treatment. However, 20 to 35% of patients may still experience residual impairments, such as impaired social functioning (9). The generally accepted hypothesis for the etiology and pathogenesis of depression is the dysfunction of the serotonin, dopamine, and norepinephrine systems in the human body. Nevertheless, some studies emphasize the significant impact of biological genetics, individual psychology, and social factors. No uniform conclusion has been reached thus far (10). For example, due to a lack of mental health resources in rural areas, rural women face a higher risk of depression, and their depressive state is concerning (11). However, other studies argue that rural women have more frequent interactions with neighbors compared with urban women, resulting in milder depressive symptoms (12). Similarly, women with higher educational levels usually have better awareness of depression and preventive knowledge, resulting in relatively better mental health (13). However, some studies have reported opposite findings (14). Furthermore, factors such as neuroendocrine changes and family support significantly influence the prevalence of depression in women at different life stages, including premenstrual, pregnancy, postpartum, and perimenopausal periods, with the depression rate during pregnancy possibly exceeding 70% (15, 16). These studies indicate that depression in women is related to biological, psychological, and social factors, but the influence of psychological and social factors is not yet clear and requires further research.

Based on this, the research focus of this study is to examine the depression state, trends, and influencing factors among women with cancer, a group particularly susceptible to depression, using data from the China Health and Retirement Longitudinal Study (CHARLS), supported by Peking University, which aims to provide references for relevant research and decision-making. This paper offers the following potential contributions: Unlike previous studies on depressive disorders in women, which mainly focus on female college students, perimenopausal women, or women in pregnancy and postpartum periods, this paper addresses the adverse effects of major diseases, specifically cancer. It focuses on the changes in depression and the influencing factors for women with cancer, a group susceptible to depression, which helps to further deepen the understanding of depression risk in Chinese women. More importantly, it helps us understand and grasp the characteristics, attributes, social standing, and development trends of women with cancer in current society. Compared to previous studies that largely rely on regional cross-sectional survey data, this study adopts nationwide household follow-up panel data from CHARLS, consistently conducted by Peking University since 2011. Such high-quality data enable a more accurate understanding of the depression characteristics and development trends of this group on a national scale. Additionally, this study incorporates the influence of individual attributes, time differences, and regional factors into a longitudinal cohort analysis, leveraging the strong statistical inference capabilities of the panel analysis method, enhancing the reliability of the research conclusions.

## 2 Materials and methods

### 2.1 Research objects and data sources

This study focuses on the changes in depression among Chinese women with cancer. The data are sourced from the China Health and Retirement Longitudinal Study (CHARLS), conducted by Peking University, which is the first nationally representative longitudinal survey of the middle-aged and older adult population in China. The survey commenced in 2011, employing a multi-stage probability proportional to size (PPS) random sampling method based on implicit stratification, which includes factors such as region, urban/rural classification, and GDP per capita. The sampling encompassed 28 provinces, 150 counties, and 450 villages across China, with a total of 10,257 households surveyed, involving 17,708 individuals (17). Follow-up surveys were conducted every 2 years. CHARLS employs stratified random sampling and conducts follow-up interviews with tens of thousands of households across the country every 2 years. The data quality is widely recognized by the academic community. For this study, baseline survey data from CHARLS in 2011, along with follow-up data from 2013, 2015, 2018, and 2020, were integrated longitudinally according to respondents’ ID codes, resulting in follow-up panel data spanning five waves. To identify the research subjects, samples of female cancer patients were screened based on the questionnaire item: “In which organ or part of your body does you have cancer? Including the origins and metastasis of tumor.”^1^ Then, by combining responses to the depression scale questions and selecting independent variables, while excluding incomplete samples, a total of 1,030 complete follow-up data entries were obtained, forming balanced short panel data with *N* = 206 and *T* = 5.

### 2.2 Selection of variables

The dependent variable in this study is the depression of Chinese women with cancer, assessed using the Center for Epidemiologic Studies Depression Scale (CES-D10). This scale is specifically designed to evaluate depressive symptoms in epidemiological studies and demonstrates good reliability and validity. It consists of 10 questions related to the emotional and behavioral experiences of respondents over the past week, encompassing various dimensions such as emotional state, sleep quality, energy level, and self-worth. Each question offers four response options: 0 = rarely or none of the time (<1 day), 1 = some of the time (1–2 days), 2 = occasionally or about half the time (3–4 days), and 3 = most of the time (5–7 days). The scores from the 10 questions are summed, with higher scores indicating a greater risk of depression and more severe depressive symptoms among female cancer patients. The total score on the scale ranges from 0 to 30, with a score of ≥10 indicating the presence of depressive symptoms. The higher the score, the greater the risk of depression in women with cancer, and the more severe the depressive symptoms.

The independent variables in this study include a range of socio-demographic characteristics of women with cancer, selected primarily from previous research. These variables encompass individual attributes (e.g., household registration type, age, marital status, educational level), disease history (e.g., ADL, hypertension, diabetes, dyslipidemia, number of outpatient visits last month, number of hospitalizations last year), health behavior variables (e.g., frequent smoking, regular visits to neighbors, regular exercise), and family characteristics (e.g., life satisfaction, family living standards, family size, health insurance). Additionally, considering the varying levels of depression among patients with different types of cancer, this paper primarily focuses on the five cancers with the highest prevalence rates. In the subsequent analysis of intergroup differences, we will specifically examine the manifestations of depression among patients with reproductive system cancers, which represent the most prevalent category. Table 1 shows the definitions and descriptive statistics of these variables. The relatively small standard deviations of the variables indicate that the data are relatively concentrated and not influenced by extreme outliers.

**Table 1:** Descriptive statistics of variables.

Variable	Definition	Mean value	Standard deviation
Reproductive system cancer	Ovarian, cervical or endometrial cancer = 1, No = 0	0.4126	0.2252
Liver cancer	Yes = 1, No = 0	0.1027	0.1117
Lung cancer	Yes = 1, No = 0	0.0924	0.0878
Breast cancer	Yes = 1, No = 0	0.0914	0.2708
Stomach cancer	Yes = 1, No = 0	0.0587	0.1074
Depressive mood	Based on the CESD-10 scale, higher scores indicate worse depression.	11.0930	6.9798
Household registration type	Rural household = 1, Urban household = 0	0.5922	0.4917
Age	Age of the respondent at the time of the survey (years)	60.7612	9.2366
Marital status	Married = 1, Others = 0	0.8398	0.3670
Educational level	Below primary school = 1, Primary school = 2, Middle school = 3, High school and above = 4	1.8126	1.0721
Activity of daily living (ADL)	Need for assistance with six daily activities (eating, dressing, bathing, toileting, transferring, and continence)	5.3136	1.2406
Hypertension	Diagnosed = 1, Not diagnosed = 0	0.3738	0.4840
Diabetes	Diagnosed = 1, Not diagnosed = 0	0.1835	0.3873
Dyslipidemia	Diagnosed = 1, Not diagnosed = 0	0.3515	0.4777
Number of outpatient visits last month	Number of outpatient visits paid by the respondent in the past month	0.4437	1.1740
Number of hospitalizations last year	Number of hospitalizations undergone by the respondent in the past year	0.7262	1.4159
Frequent smoking	Yes = 1, No = 0	0.0942	0.2922
Frequent visits to neighbors	Yes = 1, No = 0	0.2719	0.1440
Regular exercise	Yes = 1, No = 0	0.8777	0.3278
Life satisfaction	Not satisfied = 1, Slightly dissatisfied = 2, Neutral = 3, Satisfied = 4, Very satisfied = 5	3.0838	0.8415
Family living standards	Per capita annual household consumption of the respondent (CNY)	14505.7	14672.1
Family size	Number of people in the household	3.1524	1.6569
Social health insurance	Yes = 1, No = 0	0.5233	0.4997

### 2.3 Estimation method

The previous section introduced the data sources and key variable settings. This section provides further explanations regarding the estimation methods and research design. Firstly, by combining time trend tests with group difference tests, we aim to identify differences in depressive mood among various cohorts of female cancer patients, based on an overall understanding of the trends in depressive mood changes. Secondly, we select appropriate panel regression models and gradually control for a series of socio-demographic variables to examine the protective and risk factors associated with depressive moods in female cancer patients. Lastly, we conduct robustness tests to ensure the reliability of the research results by varying the dependent variable type and corresponding estimation methods (e.g., panel binary choice models, panel count models, etc.). All specific data analysis steps are performed using Stata16 software. In addition, it is important to emphasize that we obtained a complete balanced short panel data from five waves were obtained by matching cross-sectional data across different periods. Before conducting the regression analysis, it is necessary to select an appropriate panel regression model, mainly including three categories: the mixed regression model, the fixed effects model, and the random effects model.

If all women with cancer share the same regression model, that is, there are no individual effects, the mixed regression estimation model is considered, as shown in Equation (1):


(1) $$MH_{\mathrm{it}}=\alpha +\beta \times X_{\mathrm{it}}+\delta \times \nu_{i}+\mathrm{Pro}v_{i}+\varepsilon_{\mathrm{it}}$$

Where $MH_{\mathrm{it}}$ represents the depression state of a woman with cancer i during period t ; $X_{\mathrm{it}}$ is a set of time-varying socio-demographic characteristic variables corresponding to the woman; $\nu_{i}$ is a set of non-time-varying socio-demographic characteristic variables corresponding to the woman; $\mathrm{Pro}v_{i}$ represents province-specific effects that do not change over time; and $\varepsilon_{\mathrm{it}}$ is a random disturbance term. In this study, we mainly focus on the magnitude and significance changes of the β and δ values of the independent variable coefficients.

However, in real life, the socio-demographic characteristics of each woman with cancer differ, meaning not all individuals share the same regression model, and individual effects do exist. Additionally, individual heterogeneity may manifest in different time trends, such as varying income growth rates among women with cancer in the follow-up panel data across five waves. In this case, a stochastic trend model should be considered (including two types: fixed effects and random effects), as shown in Equation (2):


(2) $$MH_{\mathrm{it}}=\beta \times X_{\mathrm{it}}+\delta \times \nu_{i}+u_{i}+\gamma_{i}\times t+\mathrm{Pro}v_{i}+\varepsilon_{\mathrm{it}}$$

Where $\gamma_{i}\times t$ represents the time trend of an individual, and other variables are the same as in Equation 1. If u_t is uncorrelated with the explanatory variables { $X_{\mathrm{it}}$ , $\nu_{i}$ }, a panel random effects model (*RE*) is used for estimation; if $u_{i}$ is correlated with the explanatory variables { $X_{\mathrm{it}}$ , $\nu_{i}$ }, a panel fixed effects model (*FE*) is used for estimation.

The specific model selection is mainly based on the results of the F-test and Hausman test.

## 3 Result analysis

### 3.1 Correlation test

An extended ordinary least squares (*OLS*) panel model is employed in this study for regression analysis. To ensure the unbiasedness of the estimates, we tested the correlation coefficients of the independent variables. The Pearson and Spearman correlation coefficient tests indicate that the absolute values of the correlation coefficients for all independent variables range between 0 and 0.4. Based on this, we can preliminarily conclude that there is no “strict multicollinearity” among the variables. Furthermore, the variance inflation factor (*VIF*) test results show that the maximum VI*F* value for all variables is 1.52, which is far less than the threshold of 10. Therefore, we reject the hypothesis of “multicollinearity,” ensuring the unbiasedness of the regression analysis.

### 3.2 Characteristics of changes in depression

Using the responses to the depression scale items from five waves of follow-up data in CHARLS, we calculated the depression scores and grouped results for women with cancer in each survey year. The results are shown in Table 2.

**Table 2:** Changes in depression and group differences in Chinese women with cancer.

Depression		2011	2013	2015	2018	2020	Mean value	*T-stat*	Sample size
Full sample		10.25	10.45	10.95	12.27	11.94	11.09	—	206
Intergroup observation									
Group by urban and rural areas	Urban	8.42	9.23	10.13	10.54	11.14	9.89	−4.62^***^	84
	Rural	11.50	11.57	11.82	13.46	12.50	11.92		122
Group by educational level	Below primary school	11.39	11.70	12.12	13.24	13.53	11.99	−4.86^***^	116
	Primary school	9.52	9.58	11.18	12.46	12.88	11.79	−1.36^**^	31
	Middle school	8.12	8.82	9.89	10.19	10.31	9.23	3.81^***^	36
	High school and above	7.41	7.75	8.29	8.27	8.13	8.23	4.71^***^	23
Group by disease category	Reproductive system cancers	11.17	11.31	12.00	12.78	12.19	11.57	−1.85^**^	85
	Non-reproductive system cancer	9.60	9.87	10.21	11.91	11.60	10.76		121
Group by region	Western region	11.02	11.40	11.63	14.18	12.86	12.32	−3.38^***^	54
	Central region	10.47	10.61	11.45	12.38	11.87	11.39	−1.01^**^	72
	Eastern region	8.31	9.53	9.87	10.88	11.39	9.99	4.05^***^	80

T-stat refers to the test for the mean difference between groups, with educational level and region variables included as dummy variables in the test; *p < 0.1, **p < 0.05, ***p < 0.01.

Table 2 reports the overall depressive state of the follow-up samples across the five waves. The results of the time trend test indicate that the trend of changes in the depressive state of the follow-up sample is statistically significant (*APC* = 75.24%, *p* = 0.000). Specifically, the mean depression scores for the full sample gradually increased from 2011 (score of 10.25), reaching a peak in 2018 (score of 12.27). After that, the depression score saw a slight drop, but by the fifth wave of follow-up in 2020, the score was still 1.69 points higher than in 2011. This indicates that the depressive state of women with cancer initially worsened over time, then slightly improved, but overall, it has deteriorated. One reason is that cancer, as a critical illness, has uncertain therapeutic effects that can easily trigger negative emotions such as anxiety and fear, leading to excessive psychological stress in patients over the short term. Also, the disease itself severely damages physical health and induces perceived pain, which gradually affects closely related mental health, further amplifying the risk of depression. Therefore, the depressive state of cancer patients often worsens in the short term. However, individuals have a long-term adaptive ability to their environment. Over time, women with cancer generally improve in their understanding and acceptance of their illness. Once this understanding reaches a certain level, short-term emotional fluctuations begin to level off, resulting in an improvement in their depressive state.

**Figure 1:**
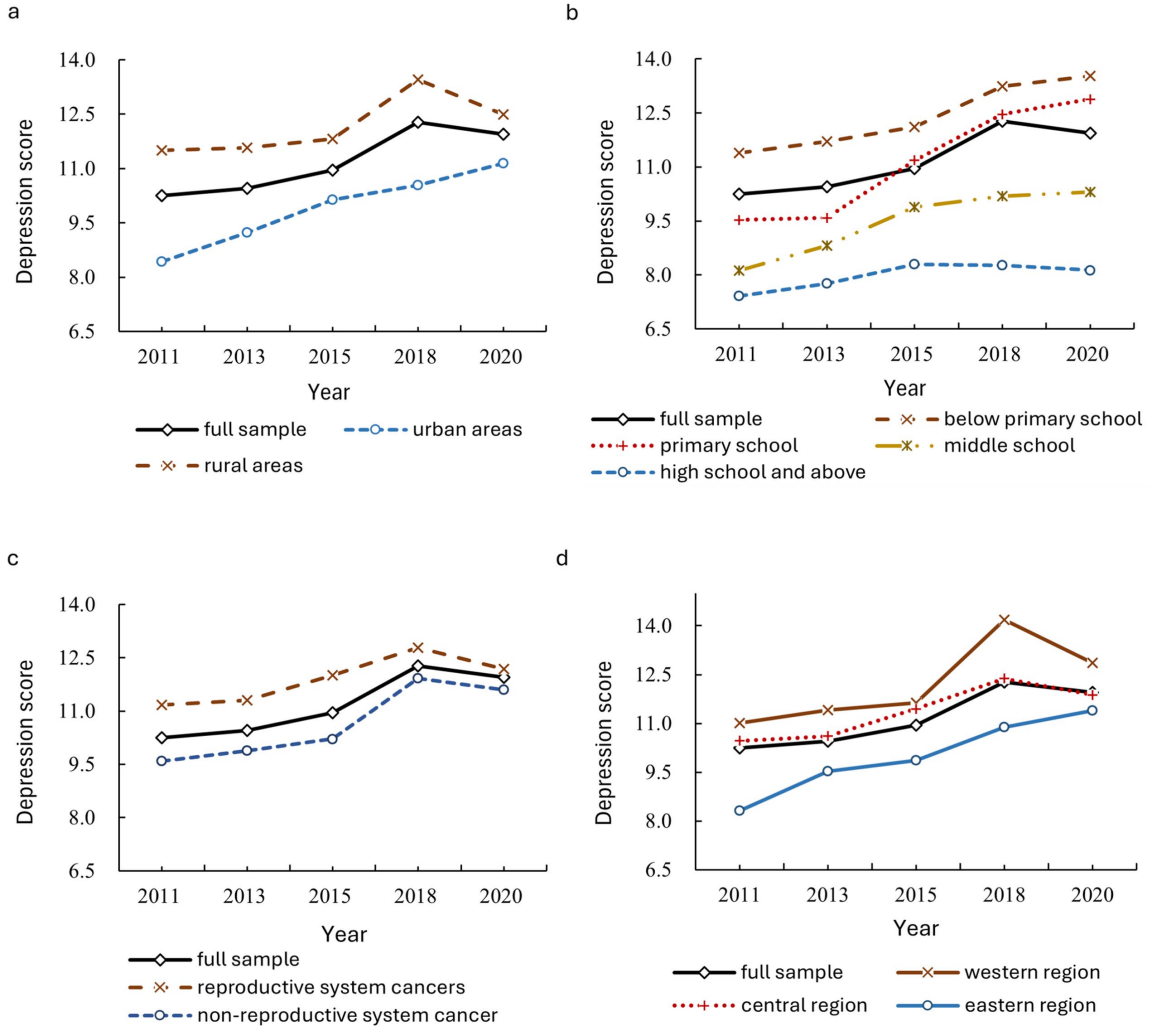
Trends in depression changes among women with cancer, categorized by urban and rural areas, educational level, disease category, and region.

Table 2 also reports the depression scores of women with cancer, categorized by urban and rural areas, educational level, disease category, and region. All statistics (*T-stat*) of the test for differences between these groups are significant at the 5% or 1% confidence level, indicating significant differences in depression among women with cancer across urban and rural residency, educational levels, disease categories, and regions. To provide a clearer illustration of these intergroup differences and their characteristics, Figure 1 displays trend charts of depression changes across the different groups. Among them, Figure 1A shows the results grouped by urban and rural areas, Figure 1B shows the results grouped by education levels, Figure 1C shows the results grouped by disease category, and Figure 1D shows the results grouped by region. Both Table 2 and Figure 1 show that overall depression scores increased during the sample period for both urban and rural women with cancer, with rural women consistently having higher scores than urban women. Unlike the continuous increase observed in the urban group, the depression score for the rural group peaked in 2018 and then declined. These findings indicate that the overall depressive state of women with cancer in both urban and rural areas worsened, with women with cancer in rural areas showing a significantly higher risk of depression and an earlier turning point in their emotional state than those in urban areas. This is primarily because rural residents, compared to their urban counterparts, generally lack awareness of depression. Social stigmatization further exacerbates the issue, leading many women with cancer in rural areas to intentionally ignore or conceal their emotional abnormalities (18), so their depression cannot be properly channeled and gradually worsen. Eventually, severe consequences force them to seek treatment and other interventions, resulting in an earlier turning point in emotional improvement.

Similarly, the results for groups by educational level and region (Figures 1B,D) show that women with cancer in the eastern region and those with higher educational levels exhibit better mental health, while those in the western region and those with educational levels below primary school show worse mental health. This disparity is attributed to the higher economic and social development levels and the greater dissemination of basic health knowledge in the eastern region. Therefore, women surveyed in the eastern region show higher levels of awareness, attention, and preventive consciousness regarding depression risks compared with those in the central and western regions. Additionally, patients with higher educational levels generally possess better external perception abilities, adaptive skills, and sensitivity to depression. They are more vigilant about maintaining their physical and mental health (19) and have relatively greater economic and social resources to prevent and resolve depression risks, resulting in a lower risk of depression. Furthermore, as shown in Figure 1C, patients with reproductive system cancers exhibit significantly more severe depression compared to those with non-reproductive system cancers. This may be attributed to estrogen secretion disorders caused by reproductive system cancers (e.g., uterine fibroids) or heightened concerns about fertility risks during reproductive age.

### 3.3 Baseline regression analysis

Since this study uses balanced short panel data, selecting an appropriate panel regression model is essential before conducting regression analysis. Table 3 reports the test results for model selection. The -values of the FtestandLMtest are far less than 0.01, leading to the rejection of the null hypothesis and indicating that the panel mixed regression model is not suitable. Instead, a fixed or random effects model should be considered. Furthermore, the statistics of Hausmantest did not pass the significance test, with a P -value close to 1, resulting in the acceptance of the null hypothesis that “the random effects model is superior to the fixed effects model.” Therefore, the random effects model is used for analysis in this paper.

**Table 3:** Test results for panel regression model selection.

Test method	Null hypothesis	Statistics	*p*-value
*F* test	The mixed effects model is superior to the fixed effects model	4.49^***^	0.0000
*LM* test	The mixed effects model is superior to the random effects model	108.62^***^	0.0000
*Hausman* test	The random effects model is superior to the fixed effects model	26.13	0.9555

*p < 0.1, **p < 0.05, ***p < 0.01.

Table 4 presents the estimated results of the factors influencing depression in Chinese women with cancer. Model (1) in Table 4 presents the full-variable regression results of the random effects model. Given the fundamental reality in China where regional economic and social development are relatively unbalanced, the impact of the same factor on the depression of women with cancer may vary due to provincial differences in resource endowments, institutional policies, and access to medical services. Furthermore, this impact may change over time.^2^ To minimize disturbances in the estimation results, Model (2) controls for provincial effects, while Model (3) further controls for potential time effects by introducing the year as a dummy variable. For comparison, column (4) provides the regression results based on the two-way fixed effects model. The results show that, by successively controlling for provincial effects and time effects, or by adopting the two-way fixed effects model, the influence of variables such as individual attributes, disease history, health behaviors, and family characteristics on the depression of women with cancer largely passed the significance test, with very stable coefficient signs. This indicates that the depression of women with cancer is closely associated with their socio-demographic characteristics. Specifically:

**Table 4:** Estimated results of the factors influencing depression in Chinese women with cancer.

Variable	Panel random effects model			
	Full-variable regression	Controlling for provincial effects	Controlling for time effects	Two-way fixed effects model
	(1)	(2)	(3)	(4)
Household registration type	0.994^***^(0.368)	0.712^***^(0.253)	0.603^***^(0.218)	0.859^*^(0.328)
Age	−0.077^***^(0.018)	−0.057^***^(0.019)	−0.067^***^(0.019)	−0.095^***^(0.016)
Marital status	−2.928^***^(0.386)	−2.476^***^(0.316)	−2.490^***^(0.285)	−2.800^***^(0.386)
Educational level	−1.099^***^(0.192)	−0.963^***^(0.202)	−0.971^***^(0.207)	−1.015^***^(0.173)
Activity of daily living (ADL)	−1.500^***^(0.256)	−1.379^***^(0.258)	−1.314^***^(0.246)	−1.440^***^(0.236)
Hypertension	−0.252(0.304)	−0.549(0.369)	−0.552(0.354)	−0.057(0.314)
Diabetes	0.864^**^(0.416)	1.106^***^(0.400)	0.912^**^(0.426)	0.778(0.462)
Dyslipidemia	0.679^***^(0.181)	0.508^**^(0.216)	0.632^***^(0.222)	0.805^**^(0.219)
Number of outpatient visits last month	0.407^***^(0.095)	0.338^***^(0.121)	0.343^***^(0.115)	0.352^**^(0.099)
Number of hospitalizations last year	0.439^***^(0.123)	0.416^***^(0.146)	0.443^***^(0.141)	0.430^**^(0.126)
Current smoking	1.188^*^(0.689)	1.682^**^(0.783)	1.744^**^(0.770)	1.444^*^(0.662)
Frequent visits to neighbors	−5.995^***^(1.036)	−6.961^***^(0.563)	−7.196^***^(0.544)	−6.686^***^(0.762)
Frequent exercise	−0.720(0.579)	−1.047^**^(0.410)	−1.067^***^(0.404)	−0.943(0.515)
Life satisfaction	−3.087^***^(0.136)	−3.076^***^(0.167)	−3.125^***^(0.181)	−3.145^***^(0.137)
Family living standards	0.476^**^(0.200)	0.485^***^(0.136)	0.341^**^(0.139)	0.333(0.198)
Social health insurance	0.647(0.395)	0.723^*^(0.416)	0.608(0.464)	0.613(0.377)
Family size	−0.265^**^(0.103)	−0.322^***^(0.119)	−0.255^**^(0.110)	−0.244(0.116)
Constant term	32.93^***^(2.568)	28.89^***^(0.762)	29.94^***^(2.986)	35.12^***^(2.506)
Provincial effects	No	Yes	Yes	Yes
Time effects	No	No	Yes	Yes
Observed value	1,030	1,030	1,030	1,030
R^2^	0.291	0.421	0.427	0.383
Wald test value/F value	406.225^***^	718.315^***^	733.070^***^	14.620^***^

^*^p < 0.1, ^**^p < 0.05, ^***^p < 0.01; robust standard errors in parentheses.

From the perspective of individual attributes, the influence coefficient for household registration type is significantly positive, while that for educational level is significantly negative. This indicates that, compared to those in urban areas, women with cancer in rural areas face a greater risk of depression. Conversely, educational attainment acts as a protective factor against depression, consistent with the results observed in the group analysis above. Similarly, age and marital status both show significant negative correlations with depression in women with cancer. This suggests that increased age, being married, and living with a spouse can effectively alleviate depression in women with cancer. The reasons for this are as follows: Over time, patients often develop a better understanding, acceptance, and adaptability to their illness, leading to more stable depression. At the same time, the presence of a spouse provides support for disease recovery. A spouse can help detect and mitigate changes in the patient’s depression in a timely manner, and their optimism, positive attitude, understanding, and tolerance convey warmth and confidence to the patient, greatly assisting in overcoming depression.

From the perspective of disease history, numerous studies have demonstrated the positive impact of physical health on mental health. The ADL, an important measure of individual physiological health, further supports this conclusion by influencing the depression of women with cancer, that is, higher ADL scores are associated with a reduced risk of depression in individuals (20). Among common chronic diseases, this study finds that hypertension does not significantly impact the depression of women with cancer. This may be due to the simple measures available to control hypertension and patients’ long-term familiarity with managing it. On the other hand, diabetes, as an inflammatory chronic disease, requires long-term and strict dietary control, glucose monitoring, and medication, which can easily cause inconvenience in patients’ lives. Additionally, the progression of diabetes is often accompanied by comorbidities affecting other organs, and can induce stress-like reactions in the body (such as changes in cortisol activity), resulting in frequent mental stress for diabetes patients, exacerbating their depression (21). Similarly, dyslipidemia can cause endothelial dysfunction and other physical discomforts, leading to increased anxiety, irritability, anger, and other negative emotions, thus raising the risk of depression. Furthermore, it is observed in this study that higher numbers of outpatient visits or hospitalizations are detrimental to improving depression in women with cancer. Both factors serve as risk indicators for depression. This is mainly because the frequent need for outpatient visits or hospitalization implies the urgency of disease screening and treatment. A high frequency of outpatient visits within the past month or hospitalizations within the past year, to a certain extent, indicates that the patient’s condition is complex and prone to recurrence, thereby increasing their worry, anxiety, and mental stress.

From the perspective of health behaviors, the influence coefficient of frequent smoking on the depression scores of women with cancer is significantly positive, indicating that frequent smoking increases the risk of depression in these patients. This is primarily because nicotine in tobacco can cause neurotransmitter disorders in the brain, making it difficult for individuals to control their emotions (22). Moreover, long-term smoking negatively affects the efficacy of medication for cancer treatments, hinders disease recovery, and further induces or worsens depression. Conversely, frequent visits to neighbors and regular exercise, as important channels for social participation, can effectively enhance interpersonal interaction and emotional exchange. These activities not only improve the physical fitness of patients but also help release their emotional stress, allowing them to face life with a more positive attitude. Therefore, frequent visits to neighbors or regular exercise contribute to improving their depression. Regarding family characteristics, life satisfaction—defined as an individual’s overall evaluation of their living conditions—is an intrinsic emotional experience and cognitive factor closely related to subjective well-being. Therefore, it is found in this study that women with cancer who report higher life satisfaction exhibit better mental health. This study also reveals that a larger family size can improve the depression of women with cancer, as more family members may provide greater economic and emotional support.

### 3.4 Robustness test

To further evaluate the reliability of the regression results, robustness tests were conducted by changing the dependent variable in accordance with the Diagnostic and Treatment Standards of Mental Disorders (2020 Edition) published by the National Health Commission of the People’s Republic of China (9). First, based on the classification criteria of the Zung Self-Rating Depression Scale (SDS),^3^ depression scores were converted into binary variables (normal state = 0, depression = 1) using a percentage scale, which is then reintroduced into the regression as Robustness Test 1. Second, based on the classification criteria of the Patient Health Questionnaire-9 (PHQ-9),^4^ depression scores were converted into ordinal variables (no depression = 1, mild depression = 2, moderate depression = 3, moderately severe depression = 4, severe depression = 5), which is then reintroduced into the regression as Robustness Test 2. Table 5 shows the results of the robustness tests. The results show that, whether using binary categorical variables or ordinal categorical variables, and whether controlling for provincial and time effects or not, the significance levels and coefficient signs of the variables influencing the depression of women with cancer are essentially consistent with the results in Table 4, suggesting that the conclusions of this study are relatively robust.

**Table 5:** Robustness test of baseline regression.

Variable	Depression (Normal state = 0, Depression = 1)		Depression (Ordinal variables)	
	Panel binary choice model		Panel count model	
Model	(1)	(2)	(3)	(4)
Household registration type	0.265^**^(0.130)	−0.077(0.220)	0.290^**^(0.130)	0.210(0.144)
Age	−0.040^***^(0.012)	−0.036^***^(0.013)	−0.041^***^(0.011)	−0.047^***^(0.013)
Marital status	−1.027^***^(0.229)	−0.845^***^(0.256)	−1.080^***^(0.213)	−0.917^***^(0.236)
Educational level	−0.538^***^(0.101)	−0.480^***^(0.110)	−0.516^***^(0.096)	−0.457^***^(0.106)
Activity of daily living (ADL)	−0.420^***^(0.069)	−0.400^***^(0.074)	−0.475^***^(0.064)	−0.441^***^(0.068)
Hypertension	−0.268(0.207)	−0.305(0.219)	−0.203(0.198)	−0.281(0.208)
Diabetes	0.316(0.241)	0.562^**^(0.266)	0.287(0.231)	0.429^*^(0.260)
Dyslipidemia	0.368^**^(0.183)	0.345^*^(0.195)	0.320^*^(0.172)	0.409^**^(0.187)
Number of outpatient visits last month	0.123^*^(0.066)	0.084^*^(0.049)	0.133^**^(0.059)	0.107^*^(0.064)
Number of hospitalizations last year	0.145^***^(0.055)	0.135^**^(0.061)	0.162^***^(0.054)	0.135^**^(0.059)
Current smoking	0.422(0.284)	0.752^**^(0.321)	0.452^**^(0.200)	0.612^**^(0.298)
Frequent visits to neighbors	−1.889(1.314)	−2.998^**^(1.496)	−1.942(1.310)	−3.132^**^(1.500)
Regular exercise	−0.056(0.252)	−0.237(0.273)	−0.120(0.238)	−0.247(0.257)
Life satisfaction	−0.993^***^(0.110)	−1.096^***^(0.119)	−1.023^***^(0.101)	−1.130^***^(0.109)
Family living standards	0.233^**^(0.100)	0.258^**^(0.108)	0.236^**^(0.095)	0.159(0.106)
Social health insurance	0.292^*^(0.175)	0.308^*^(0.184)	0.215^*^(0.126)	0.253^*^(0.134)
Family size	−0.172^***^(0.057)	−0.206^***^(0.061)	−0.157^***^(0.053)	−0.071^*^(0.039)
Provincial effects	No	Yes	No	Yes
Time effects	No	Yes	No	Yes
Observed value	1,030	1,030	1,030	1,030
*Wald* test value	281.24^***^	330.61^***^	330.88^***^	415.03^***^
*LR* test value	29.67^***^	12.95^***^	48.29^***^	51.49^***^

^*^p < 0.1, ^**^p < 0.05, ^***^p < 0.01; robust standard errors in parentheses.

## 4 Further discussion

Globally, female cancer patients frequently experience symptoms of depression, an issue that should not be overlooked in Chinese women with cancer. Utilizing the five-phase tracking data from the China Health and Retirement Longitudinal Study (CHARLS) and the epidemiological depression scale (CES-D10), this paper assessed the depressive status of Chinese women diagnosed with cancer. The findings revealed a significant deterioration in the overall depressive mood among this population, with a depression detection rate of 57.28% in 2020, indicating a generally moderate risk of depression. On one hand, the psychological stress associated with a cancer diagnosis and the fear of survival can intensify the risk of depression. On the other hand, the side effects of cancer treatment can profoundly impact patient’ body image, easily triggering or exacerbating feelings of helplessness and anxiety (4, 23). At the same time, the lack of social support and insufficient knowledge about depression in China further exacerbate its prevalence (24). These factors collectively contribute to the high detection rate of depression and the elevated risk among women with cancer in China. This aligns with the findings of several scholarly studies (2, 6, 15), indicating that women with cancer are a vulnerable population regarding depression. Therefore, increased policy attention is warranted for this group, particularly in terms of education and screening for depression prevention and treatment. It is recommended to produce a professional, authoritative, and easily comprehensible promotional video on depression prevention and treatment to enhance public awareness. Additionally, efforts should be made to improve the early diagnostic capabilities of healthcare institutions at all levels, providing the public with both online and offline depression assessments and treatments. Furthermore, emotional state evaluations should be integrated into the routine care of cancer patients to facilitate early intervention for those suspected of having depression.

It is important to recognize that psychological resilience and the ability to resist depression vary across different population groups, and consequently, the diagnostic and treatment approaches for depressive disorders cannot be generalized (18). This study indicates that cancer patients residing in urban areas, particularly in the eastern region, with higher education levels and non-reproductive system cancers, exhibit better outcomes compared to those from rural areas, the central and western regions, with lower education levels and reproductive system cancers. In contrast to previous studies, this research highlights the intricate influence of socioeconomic factors on mental health. As noted by Gonzalez & Cruz, urban environments frequently offer greater social support and medical resources, which may significantly contribute to the disparities in depression levels observed between urban and rural women (25). Patients with elevated education levels typically demonstrate enhanced external perception abilities, self-adaptive skills, and sensitivity to depressive emotions. Furthermore, they often have access to more economic and social resources that can help prevent and alleviate the risk of depression, resulting in a lower likelihood of experiencing depressive symptoms (14, 19). However, some studies have suggested that the effect of education level on women’s depressive emotions is not significant (26, 27). Thus, while socioeconomic factors undeniably influence depressive emotions, mental health phenomena may manifest distinct characteristics and mechanisms in different regions and cultural contexts. Accordingly, the diagnosis and treatment of depressive disorders in this population must account for both urban–rural and regional differences, enhancing the understanding of the characteristics and development of depressive emotions among cancer patients in rural and central-western areas. Treatment plans should be tailored to individual demographic characteristics, including education level, cancer type, and severity of depression.

This study reveals the protective effects of various sociodemographic factors on depression in female cancer patients, including increasing age, having a spouse, good daily living abilities, frequent social visits, and regular exercise. These factors not only reflect individual quality of life and social support networks but also indicate potential intervention strategies for preventing and alleviating depressive disorders. Increasing age is associated with a reduced risk of depression in female cancer patients, likely due to the fact that older women often possess richer social relationships and life experiences (15). Additionally, their psychological resilience generally increases with age, enhancing their perception of and adaptability to the disease (28).

The presence of a spouse is also crucial for maintaining the psychological health of cancer patients; spouses are typically more attuned to their partner’s emotional fluctuations, which allows for timely identification and alleviation of worries and anxiety, thereby assisting patients in better coping with the disease and mitigating the onset of depression (29).The influence of daily living abilities on depression among female cancer patients aligns with existing research findings (11, 20), suggesting that higher daily living skills are associated with a lower risk of depression. Furthermore, social activities, such as frequent social visits and regular exercise, promote interpersonal interaction and emotional communication (3, 12). A larger family size can also provide greater economic and emotional support, effectively reducing the incidence of depression in cancer patients (24). Therefore, it is imperative to strengthen the development of social networks and familial emotional connections for female cancer patients. Psychological interventions should focus on body empathy and body image (23, 30), fostering increased empathy and care for patients’ physical and mental health. Additionally, it is essential to fully utilize the buffering effects of social support, family support, and self-emotional regulation in combating depressive emotions.

In addition, this study found that factors such as diabetes, lipid disorders, frequent visits to medical facilities, frequent hospitalizations, and smoking are significant risk factors for depressive mood. Cancer patients with diabetes and lipid abnormalities often suffer from additional organ diseases, which may trigger the body’s stress response, including alterations in cortisol activity (20). Furthermore, long-term strict dietary control not only impacts daily life but also fosters feelings of doubt, irritability, and anger, thereby increasing the risk of depression (21). Frequent outpatient visits or hospitalizations often reflect the complexity and recurrence of the patient’s condition, which further exacerbates mental stress (10). Additionally, smoking not only compromises the effectiveness of cancer treatments but also leads to disturbances in brain neurotransmitters due to prolonged tobacco inhalation (22), making it challenging for individuals to regulate their emotions, thus inducing or worsening depressive moods. Therefore, it is recommended to implement comprehensive policy measures that closely monitor the adverse effects of physiological diseases, enhance the management of chronic conditions such as diabetes and lipid abnormalities, and reduce the risk of complications. Additionally, optimizing medical processes and improving diagnostic and treatment efficiency are essential to prevent patients from exacerbating their risk of depression due to the progression of chronic diseases or frequent medical visits. Simultaneously, establishing specialized smoking cessation services that provide scientific methods and support for quitting is advisable to help patients stop smoking and improve the overall health and treatment efficacy of cancer patients.

## 5 Conclusion

The depressive emotions experienced by women with cancer in China are exhibiting a worsening trend, currently categorized as a moderate risk level for depression. Research indicates that urban women with cancer report better depressive emotional states compared to their rural counterparts. Furthermore, those residing in eastern regions, possessing higher educational levels, and diagnosed with non-reproductive system cancers fare better than individuals from central and western regions, those with lower educational levels, and reproductive system cancer patients. Additionally, factors such as increasing age, marital status, higher educational attainment, good daily living abilities, frequent social interactions, and regular exercise act as protective factors against depression in women with cancer. Conversely, conditions such as diabetes, abnormal blood lipid levels, frequent medical visits, hospitalizations, smoking, and lower life satisfaction significantly elevate the risk of depression. This paper holds public health significance by guiding early prevention and control strategies for depression among women with cancer. Moving forward, it is crucial to develop targeted mental health promotion policies for this population, emphasizing the needs of different demographic groups and regions, and to fully utilize the positive influence of socio-demographic factors in preventing and mitigating depressive disorders.

## Data Availability

The datasets presented in this study can be found in online repositories. The names of the repository/repositories and accession number(s) can be found in the article/supplementary material.

## Footnotes

^1^The CHARLS survey encompasses 20 types of cancer affecting women, including malignancies of various body tissues or organs such as the brain, oral cavity, larynx, pharynx, thyroid, lung, breast, esophagus, stomach, liver, pancreas, kidney, ovary, cervix, endometrium, colon, rectum, bladder, skin, non-Hodgkin lymphoma, and leukemia. However, while the survey includes both primary and metastatic tumors, it fails to clearly distinguish between the two and lacks statistical information regarding the duration of the disease.

^2^We posit that the influence of socio-demographic factors on the depression of women with cancer may be affected by changes over time. The reason lies in the fact that considering the numerous socio-demographic factors influencing the depression of women with cancer, there may be unobservable factors that vary over time but not between individuals, which are not controlled for in this study. Moreover, the joint significance test of the time fixed effects model shows *F* = 3.31 and *p* = 0.012, which indicates the presence of time effects to some extent. On this basis, we controlled for these time effects on the estimation results by introducing the year as a dummy variable.

^3^The Zung Self-Rating Depression Scale (SDS) has a score range of [0, 100]. According to the Chinese norm, an SDS score of less than 53 is considered normal, while an SDS score between 53 and 100 is considered depression.

^4^The Patient Health Questionnaire-9 (PHQ-9) has a score range of [0, 27], with [0, 4] = no depression, [5, 9] = mild depression, [10, 14] = moderate depression, [15, 19] = moderately severe depression, and [20, 27] = severe depression. The depression scale score interval in this study is [0, 30], so after linear transformation and rounding, ordinal variables with values from 1 to 5 were obtained.

## References

[1] Bray F Laversanne M Sung H Ferlay J Siegel RL Soerjomataram I. Global cancer statistics 2022: GLOBOCAN estimates of incidence and mortality worldwide for 36 cancers in 185 countries. CA-A Cancer J Clinicians. (2024) 74:229–63. doi: 10.3322/caac.21834, PMID: 38572751

[2] Lu J Xu XF Huang YQ Li T Ma C Xu GM. Prevalence of depressive disorders and treatment in China: a cross-sectional epidemiological study. Lancet Psychiatry. (2021) 8:981–90. doi: 10.1016/S2215-0366(21)00251-0, PMID: 34559991

[3] Schuch FB Vancampfort D Firth J Rosenbaum S. Physical activity and incident depression: a systematic review and meta-analysis of prospective studies. Am J Psychiatry. (2022) 179:109–20. doi: 10.1176/appi.ajp.2021.21010002

[4] Sebri V Pravettoni G. Tailored psychological interventions to manage body image: an opinion study on breast cancer survivors. Int J Environ Res Public Health. (2023) 20:2991. doi: 10.3390/ijerph20042991, PMID: 36833684 PMC9957299

[5] World Health Organization (WHO). Depression and other common mental disorders: global health estimates. (2017). Available at: https://www.who.int/publications/i/item/depression-global-health-estimates (Accessed May 21, 2024).

[6] Huang YQ Wang Y Wang H Liu ZR Yu X Yan J. Prevalence of mental disorders in China: a cross-sectional epidemiological study. Lancet Psychiatry. (2019) 6:211–24. doi: 10.1016/S2215-0366(18)30511-X, PMID: 30792114

[7] Fu XL Zhang K Chen XF Chen ZY. The 2023 blue book of China's mental health. (2024). Available at: http://psych.cas.cn/news/zhxw/202302/t20230224_6682792.html (Accessed May 21, 2024).

[8] Gutiérrez RL Porras SA Dunne H Andrade GN Cervilla JA. Prevalence and correlates of major depressive disorder: a systematic review. Brazilian J Psychiatry. (2020) 42:657–72. doi: 10.1590/1516-4446-2020-0650, PMID: 32756809 PMC7678895

[9] National health and family planning commission of the People's Republic of China. Guidelines for diagnosis and treatment of divine disorders. (2020). Available at: https://wjw.fujian.gov.cn/xxgk/fgwj/gjwj/202012/t20201209_5478701.htm (Accessed June 8, 2024).

[10] Zhu LJ Xu LW Kething LI. Research progress on pathogenesis of perimenopausal depression and its prevention and treatment by traditional Chinese medicine. Chin J Exp Tradit Med Formulae. (2024) 9:242–50. doi: 10.13422/j.cnki.syfjx.20240443

[11] Yang L Zong ZH. Current status and influencing factors of depression among rural middle-aged and older women in China. Chinese General Prac. (2023) 26:3091–111. Available at: https://link.cnki.net/urlid/11.5699.R.20230316.1830.056 (Accessed June 8, 2024).

[12] Chen JH Gao B Xie YC He L. Exploring the influence of social capital on depressive symptoms among female residents in Chengdu. Chinese J Health Educ. (2023) 39:206–10. doi: 10.16168/j.cnki.issn.1002-9982.2023.03.003

[13] Liao YG Zhang BY. Developmental trajectories of depressive symptoms among middle adulthood: based on growth mixture modeling. J Psycholog Sci. (2024) 2:300–7. doi: 10.16719/j.cnki.1671-6981.20240206

[14] Patria B. The longitudinal effects of education on depression: finding from the Indonesian National Survey. Front Public Health. (2022) 10:e1017995. doi: 10.3389/fpubh.2022.1017995, PMID: 36339172 PMC9632623

[15] Tomita A Burns JK. A multilevel analysis of association between neighborhood social capital and depression: evidence from the first south African national income dynamics study. J Affect Disord. (2023) 144:101–5. doi: 10.1016/j.jad.2012.05.066, PMID: 22858263 PMC3513630

[16] Yang ZT Wen X Ye QY. The detection rate of depression during childbearing process among Chinese women: a meta-analysis. Chinese J Dis Control Prevent. (2023) 12:1475–9. doi: 10.16462/j.cnki.zhjbkz.2023.12.018

[17] Zhao YH Hu YS Smith JP Strauss J Yang GH. Cohort profile: the China health and retirement longitudinal study (CHARLS). Int J Epidemiol. (2014) 43:61–8. doi: 10.1093/ije/dys203, PMID: 23243115 PMC3937970

[18] Liang HX Huang RF Wang YH. Association of anxiety symptom and self-stigma with allostatic load in patients with rare diseases. Chinese J Preven Control of Chronic Dis. (2023) 2:110–6. doi: 10.16386/j.cjpccd.issn.1004-6194.2023.02.006

[19] Andersen BL Lacchetti C Ashing K Berek JS Berman BS Bolte S. Management of anxiety and depression in adult survivors of cancer: ASCO guideline update. J Clin Oncol. (2023) 41:3426–53. doi: 10.1200/JCO.23.00293, PMID: 37075262

[20] Zhang SS Wang WL Li LL. Study on the mediating effect of activities of daily living between arthritis and depressive symptoms in the older adult. Modern Prev Med. (2024) 1:123–55. doi: 10.20043/j.cnki.MPM.202308135

[21] Jeffery A Bhanu C Walters K Wong ICK Osborn D Hayes JF. Polypharmacy and antidepressant acceptability in comorbid depression and type 2 diabetes: a cohort study using UK primary care data. Br J Gen Pract. (2022) 73:e392–8. doi: 10.3399/BJGP.2022.0295, PMID: 37105749 PMC9923766

[22] Kiviruusu O Berg N Piirtola M Viertiö S Suvisaari J Korhonen T. Life-course associations between smoking and depressive symptoms: a 30-year finnish follow-up study. Nicotine Tob Res. (2024) 26:843–51. doi: 10.1093/ntr/ntae012, PMID: 38243907

[23] Sebri V Durosini I Pravettoni G. How to address the body after breast cancer? A proposal for a psychological intervention focused on body compassion. Front Psychol. (2023) 13:1085837. doi: 10.3389/fpsyg.2022.1085837, PMID: 36698594 PMC9868453

[24] Chen X Yang Y. The role of social support in alleviating depression symptoms: evidence from female cancer survivors in China. J Affect Disord. (2024) 327:137–44. doi: 10.1016/j.jad.2023.02.012, PMID: 36754090 PMC9899704

[25] Gonzalez JM Cruz A. Comparison of depression symptoms between urban and rural populations: a systematic review. J Affect Disord. (2022) 311:463–71. doi: 10.1016/j.jad.2022.05.055 35580695

[26] Wei J Yi L Xie HH. Education and mental health: evidence and mechanisms. J Econ Behav Organ. (2020) 180:407–37. doi: 10.1016/j.jebo.2020.09.032, PMID: 39743835

[27] Pei RJ Li L Xing YA. Analysis of the differences in depression tendencies and influencing factors among urban and rural older adult populations. Chinese J Public Health Manag. (2019) 35:297–300. doi: 10.19568/j.cnki.23-1318.2019.03.003

[28] Du XM Deng GY Yang ZT. Analysis of the risk and influencing factors of perinatal depression in women of different age groups. Chinese J Preven Med. (2024) 25:300–6. doi: 10.16506/j.1009-6639.2024.03.008

[29] Yang JL Li YH Li CH. Research progress on peer support in the recovery of patients with mental disorders. Military Nurs. (2024) 41:90–6. Available at: https://kns.cnki.net/kcms2/article/abstract?v=Dm4VI7mKrXM7lvcLWmtwbOEQH0sxp8IxH8J-fp1M5V5qDMdbKLg0LN4bKsHlp9Emv-JAvd9ryTcvA9w8TjTm7MRZ33g0tkO-_FwJdhZ_kkMyjjFQrAexvlq5qVla7_p3I2-HnfPbKhlkVl2t7pBnZlQqgM6xEwQ--xYpBosvjaqQ3bROFEx-DasgedDn0DVV6UtEZYz-hdk=&uniplatform=NZKPT&language=CHS (Accessed June 8, 2024)

[30] Kirby JN. Compassion interventions: the programmes, the evidence, and implications for research and practice. Psychol Psychother. (2017) 90:432–55. doi: 10.1111/papt.12104, PMID: 27664071

